# Supposed Demoniacal Possession

**Published:** 1849-07-01

**Authors:** 


### SUPPOSED DEMONIACAL POSSESSION.

[The following article was placed in our hands by the author himself for publication. We have endeavoured to establish, to the writer's satisfaction, that he has formed an erroneous notion of the imaginary influence exercised over him. It has been our object to prove to him that he has been labouring under an illusion, the result of a disturbed condition of the functions of the brain and nervous system. It is difficult, however, to remove the impression that the writer has, that he is the subject of demoniacal agencies. We have had the pleasure of seeing the party on several occasions, and on all other points he appears to be a rational, sensible, and intelligent man. We print the history of his own sensations without the slightest alteration of our own. It will be of interest to the psychological philosopher. The author has promised to give us a further history of his life in connexion with his "illusions," as we designate them. If he so favour us, the article will appear in the next number of the journal.—Editor.]

*To the Editor of the "Journal of Psychological Medicine."*

Sir,—It was my intention, some time since, to write a short account of the sufferings I had experienced, for several years past, from the possession of evil spirits; but in consequence of having been constantly pitied or smiled at, and having met with no one who would sympathize with me, whenever I broached such a notion, and instanced myself as a proof of the existence of such spirits, I had nearly forsaken my resolution, until I found that the question, whether demoniacal possession ceased or not at the period of our blessed Lord's ascension, had really become a matter of importance and doubt in the church.

Unacquainted as I am with theological discussions, and wholly unused to argumentative composition, I am at a loss in what manner to set about an explanation on the subject required. May I trust that by commencing with a slight sketch of my life, rendering some detail of the affliction I have undergone, with the authority of the New Testament, I may create the thought and establish the impression that even at this time the visitation of mortals by evil spirits is still permitted by the Most High.

I am induced to enter into a narrative of my own life and feelings, in order to show that I am not a person likely to be influenced by superstition or bigotry; and by thus developing myself, I hope to gain the confidence and conviction of the reader, although to me it is a disagreeable task to be egotistic.

I was born in the East, my grandfather and father both being officers of some note in the Company's service, and was brought by my parents to England for the customary purpose of education, and on their return to India was left by them here, under the charge of a brother officer of my grandfather, who was then retiring from the service. He was a very enlightened and good man, and albeit a Roman Catholic, brought me up to the religion of my parents, which was that of the Established Church. My father had, however, I believe, previously to his entrance into the army, belonged, as did most of his relations and connexions, to the Society of Friends.

Whilst I remained with my guardian, the only book he placed in my hands on religious topics was the Bible, from which he said I ought to be able to form my own religion, irrespective of the tenets of any sect. He would not hold any theological arguments with me; but whatever simple explanations I required, he was ready to give, without advancing his own Roman-catholic doctrines.

My father having died in India shortly after his return there, before I was eight years old, and my mother continuing to reside there, I remained under the sole care of my guardian during my minority, in the course of which I was placed at several good schools in the neighbourhood of the metropolis, where I obtained what little learning I am possessed of.

Expecting to get an appointment in the Indian army, and having been disappointed in consequence of my hue, and the prejudice then entertained by the government to native officers, I chose a liberal profession, and at an early age was declared competent to follow it, which, as my mother was then still in India, I intended to enter into there; but on account of her return to England, relinquished such idea, and commenced business in this country, which, having carried on for some years with success, induced me to marry, and I was blessed with a good partner and several fine children. The profits from my profession still continuing on the increase, I entered into some money speculations, which caused me a little anxiety and some pecuniary embarrassment, but I retained all my usual buoyancy of spirit.

It was then, whilst taking a quiet walk one evening, far from the busy hum of men, about five years since, I heard the sound of voices near me, speaking of me. I looked in every direction, but could not discover any one; I got over some banks, thinking that, probably, the persons might have been concealed from view by them; but no human creatures were there. I walked away from the spot, still the voices pursued me. I mixed with the thickest of the throng in the metropolis; the voices still continued to haunt me, and the words then uttered were—"Who is he?—do you know who he is?" The response was—"He is Satan's own." These words seemed continuously to proceed from the persons I passed. I crossed and recrossed the bridges; still the same voices followed me. Every one appeared to ask the same or a like question, and there was a similar reply. Other queries and answers succeeded these, relating to my walking—for my pace was very rapid, as I trusted to escape the notice or recognition of the passers-by; but the "Devil's Own" was either whispered or shouted to me, apparently by almost every one; and those from whom the sounds did not emanate, appeared hastily to get out of my way, or, in my imagination, shrunk from me with looks expressive of surprise. No doubt, however, that my strides were those of a possessed person, and caused those I met or overtook to make ample space for me.

The whole night did I thus perambulate London and its environs, occasionally dozing as I stood still for a few minutes; and in this manner I twice accomplished the circuit of the great city, vainly hoping that daylight would end my illusion. Such hope was, indeed, vain, and I must mention, that not merely the "devil's own" was sounded in my ears, but observations and conversations relating to me incessantly occurred. Yet was I perfectly in my senses. I went to the place in which the sounds first reached me, and examined it and the neighbourhood minutely; of course I could not discover any human power to account for them. I then began to think of animal magnetism; it was a subject on which I had thought little before, but not being able in any other way to fathom the mystery, the consideration of it and its effects occupied my mind, and I reasoned that I might have been magnetized by a nautical compass, which had belonged to my father, and that I had constantly carried about me for a considerable length of time. The voices loudly and clamorously spoke of all my misdeeds, and taxed me with sins of which I had not been guilty, and I was dared to meet the parties who charged me with such and with other crimes. I did, accordingly, go to a friend of mine, who is now dead, and told him how I had been affected, and that I wished him to be present to hear the voices, if he could, and the charges to be made against me, which I was anxious to deny, or to admit, as the circumstances had been. Several voices then made various accusations against me, and I appeared to be put on a regular trial. I replied to the charges by my thoughts, without speaking, but occasionally my tongue could not refrain from moving within my lips to express my thoughts, without, however, giving utterance to them. One of the voices was remarkably clear and loud. It appeared to be that of a being of authority in conversation with another, and although slightly favourable in his expressions of my good conduct throughout life, yet strong and severe were his animadversions on my bad thoughts and actions; and here everything I had said, or done, or omitted was elucidated instantly; hidden motives, and thoughts, and actions, were unravelled, to my great astonishment, and my heart and brain seemed completely laid open. All was written down, or directed so to be, and the next day was appointed for a further examination.

I asked my friend repeatedly during this apparent trial, if he heard any voices. He told me he did not. I mentioned to him what was now and then said to me, and of me. I smiled at myself, for I knew I was only in a room, and that it was impossible for any worldly being to speak or to communicate with me except my friend. I looked at him—he was deeply engaged in writing: could there be any ventriloquism in my case? I knew that my friend was not thus gifted. Besides, the voices were with me before I saw him that day. What could have occasioned the sensation of sound I had experienced? —the direct appeal to my heart and brain? I was entirely in my senses, and reasoned on the absurdity of my harbouring any opinion contrary to my own received notion of the ordinary laws of nature. I began to think of mesmerism—of clairvoyance. I had been sceptical on these subjects. Could I have been mesmerised? How long would the mesmeric symptoms last? I had a strong mind—how, then, could I have been affected by any one? The more I thought, the less could I account for the extraordinary ordeal to which I was subjected. I did not believe in evil spirits. What I had read in the Testament relating to evil spirits, I had always construed as having reference to madness or derangement of intellect, that had been cured by our Saviour. I did not believe in the commonly received notions of hell —fire and flames had no terrors for me, nor have they now. The torment that I considered awaited us after judgment, was the sting of our own consciences—the reflection that we were justly debarred from the presence of God—the constant remembrance of our misdeeds —the bitterest, the most poignant remorse.

To return. After this seeming trial, the remainder of which I told my friend would be deferred till the morrow, when I would see him again, I left him. The voices still continued to follow me. That night I also walked about, for I did not wish to return home with the words, "The Devil's Own," written, as it almost appeared to me, on my back, or with the sounds of those words preceding me, or announcing me to every one.

I did not, nor do I put any faith in fatality. I have always been in the habit of considering, that man would be an irresponsible being in connexion with fate—that if he were fated or obliged to do any act, he would certainly not be answerable or accountable for it, and for this reason I was an advocate for free will. This did not, of course, exclude the notion of the predisposing gift of grace influencing us towards what was good and holy; but it would still leave us free thought and liberty in our actions. My mind now, however, felt fettered, contrary to my will—my thoughts were carried into channels that I not only did not desire, but that I studiously and with all the energy in my power, endeavoured to prevent them rushing into the stream of. I appeared in the grasp of superior beings.

The next day I went prepared for another examination, but I was not again put on my trial. The parties seemed partly satisfied with my mental engagement of compensation, as far as I had the ability, of any persons I might have injured in thought, word, or deed. My friend then induced me to lie down to compose myself. I returned home. Still the voices followed me, and imagination can but slightly picture the constant wearying sounds of remarks on me—speeches to me—alternately on my actions and thoughts, bringing all that I ever did, or said, or thought to recollection. In the day-time I did not feel the annoyance so much, on account of the variety of things and persons I saw, and the occupation I had; but in the stillness of night, the torments I endured were unutterable—indescribable. The hellish sounds; the dreadful impieties that were spoken of—that were foisted on me; the horrible exclamations and imprecations which I distinctly heard; the fiendish crimes proposed—were beyond conception; were such as man, and much more a Christian, would shudder at the bare mention of. Day after day, night after night, was I subjected to this visitation, not at intervals, but continually; indeed, each moment of my life was embittered by these sounds; and the only respite I had, was when nature was wholly exhausted, and two or three, or sometimes four, hours' repose were absolutely necessary to renew my existence the following day, under such complicated sufferings. When I attempted to pray, I could not, for the jeering, and laughter, and impious reflections that were obtruded on me. I tried to read, but could only get through a few short sentences at a time, and those, owing to the voices, I could hardly retain in my recollection. I asked forgiveness of those I had in any way injured; I read the New Testament, but I seemed, almost insensibly to myself, to omit all, except our Saviour's words, which I read aloud; these gave me more consolation than anything else. I wished to have prayers read to me, for I thought the evil spirits might quit me in the presence of a clergyman. One kindly came; I could not pray, and was obliged to tell him so. I felt that I could not kneel. His prayers soothed me slightly, but the spirits remained.

For change of scene, and hoping I should get free from the voices, I went twice to France. I tried the sea coast in England, and all kinds of amusements, and also the effect of living very well, thinking, my nerves might be improved by a still more generous regimen than I had ever been accustomed to. These having no effect, I had myself cupped, and entirely altered my diet, living chiefly on vegetables, and avoiding all fermented liquors. Nothing, however, made any difference in my sensations. The sounds accompanied me everywhere, and I still continued the prey of the evil spirits. I could plainly distinguish about seven voices; two of them struck me as the voices of females; one of these sometimes spoke in over-soothing, complaisant accents to me, but these were generally used only to turn me to ridicule afterwards. The seven voices remained with me many months, when three left me, and four continued to torment me for nearly a couple of years; and since then I have only had two, a male and female, who have gradually less and less annoyed me for the past year. It is now five years and four months that I have had this visitation from God, and although I have no faith in dreams, yet most singularly I dreamt of my father's death about the time it occurred, and I have not dreamt of him since, until the beginning of this month of September, when I dreamt that I saw him interceding with God for the ceasing or suspension of my sufferings from evil spirits, and, strange to say, I have not been troubled by them since, although I still fancy I hear a slight buzzing in my ears, from their having been so many years my constant companions.

It would take me many hours to express all the machinations of the evil spirits that have possessed me during so lengthened a period as five years and upwards; but by the power and mercy of God, through the merits of my Saviour, I was enabled to bear the sufferings to which I was exposed, and also partly to resist the temptations to which I was subjected; and, as I said before, I did not previously believe in evil spirits, but since my affliction, I have had evidence in my own person, fully sufficient to satisfy me, that they are permitted to dwell in persons, or to attend persons in this world, for the purpose of proving them and of tormenting them if they sin.

I can now readily understand the dreadful agony sustained by those possessed of devils mentioned in the Holy Scripture, and I would humbly venture to account for our Saviour's temptation in the wilderness, when having fasted forty days and forty nights, he was afterward an hungered, and the tempter came to him and said, "If thou be the Son of God, command that these stones be made bread." Here I have little doubt that the devil did not really appear bodily, as it may be termed, but spiritually suggested or said those words to our Saviour without making his appearance. "Then the devil taketh our Saviour up into the holy city, and setteth him on a pinnacle of the temple." I do not know what the Hebrew word that is translated "pinnacle" implies, but if, probably, it was a summit of the temple that was ascendible, it could be accounted for, as it would then be that, at the instigation of the devil, our Saviour went into Jerusalem, and to this elevated part of the temple. Again, it would be similar as regards our Saviour being "carried up into an exceeding high mountain." The evil one, in my own case, has never appeared to me. I have had wealth and power offered by the evil spirits to me, if I would give myself up to or worship Satan; but by the blessing of God, through the mediation of my Saviour, I was enabled to resist the temptation.

That in the time of our Saviour evil spirits or devils were common there can be no question, as they are repeatedly referred to throughout the writings of the evangelists. And there can be as little doubt that devils or unclean spirits remained after our Saviour's ascension, as they were cast out by the apostles, as appears in the Acts, v. 16; viii. 7; xvi. 18; xix. 12. Not having quitted the world, therefore, at the period of our Saviour's ascension, it is not by any means probable that evil spirits deserted it on the death of the apostles; and there is no reason to suppose that they are not now still allowed to visit the earth. I was wholly an unbeliever in this respect, but I now entertain no doubt on the subject, from my past long-tried experience; and it strikes me that many persons who are considered and pronounced deranged, are really, instead, possessed by evil spirits. It may be said that I may myself be in a state of derangement. To this I would oppose these facts—that I do not pretend to having had any ocular demonstration of any spirit, nor have I had any distorted visions or ideas. I have not spoken incoherently, nor have I acted contrary to rationality; but I have always been blessed with my senses, notwithstanding this heavy calamity of evil possession, with which it has pleased God to visit me, and which he has now been graciously pleased to remove from me. May all others, similarly afflicted, experience, in like manner, the mercy and goodness of God.—I am, Sir, your obedient servant,

